# Therapeutic target of high fresh frozen plasma to red blood cell ratio in severe blunt trauma

**DOI:** 10.1186/s13054-025-05678-z

**Published:** 2025-10-16

**Authors:** Gaku Fujiwara, Kosuke Inoue, Wataru Ishii, Tadashi Echigo, Shoji Yokobori, Naoto Shiomi, Naoya Hashimoto, Shigeru Ohtsuru, Yohei Okada

**Affiliations:** 1 Department of Neurosurgery, Kyoto Prefectural University of Medicine, 465 Kajii-cho, Kamigyo-ku, Kyoto, Japan; 2 Department of Neurosurgery, Japanese Red Cross Society Kyoto Daini Hospital, Kyoto, Japan; 3 Department of Social Epidemiology, School of Public Health, Graduate School of Medicine, Kyoto University, Kyoto, Japan; 4 Hakubi Center for Advanced Research, Kyoto University, Kyoto, Japan; 5 Department of Emergency Medicine and Critical Care, Japanese Red Cross Society Kyoto Daini Hospital, Kyoto, Japan; 6 National Health Insurance Yamato Clinic, Kagoshima, Japan; 7 Department of Emergency and Critical Care Medicine, Nippon Medical School, Tokyo, Japan; 8 Department of Critical and Intensive Care Medicine, Shiga University of Medical Science, Shiga, Japan; 9 Department of Primary Care and Emergency Medicine, Kyoto University, Kyoto, Japan; 10 Department of Preventive Services, School of Public Health, Kyoto University, Kyoto, Japan; 11 Pre-hospital and Emergency Research Centre, Health Services Research and Population Health, Duke-NUS Medical School, National University of Singapore, Singapore, Singapore

**Keywords:** Transfusion, Blood product ratio, Fresh frozen plasma, Multiple trauma, Blunt trauma, Massive transfusion, Coagulopathy, Causal forest, Machine learning, Heterogeneous treatment effect

## Abstract

**Background:**

To assess heterogeneous treatment effects of high fresh frozen plasma (FFP) to red blood cell (RBC) transfusion ratios in patients with severe blunt trauma and to identify subgroups that derive the greatest survival benefit.

**Methods:**

This multicenter retrospective cohort study used data from the Japan Trauma Data Bank (2019–2023). Adults with severe blunt trauma (Injury Severity Score ≥ 16) who received transfusions were included. Patients were categorized into high-FFP (FFP:RBC > 1) and low-FFP (FFP:RBC ≤ 1) groups. A causal forest machine learning model was applied to a derivation cohort (2019–2021) to estimate conditional average treatment effects (CATEs) and identify subgroups with the highest predicted benefit. Findings were validated in a separate cohort (2022–2023).

**Results:**

Among 6,679 patients, in-hospital mortality was 23.3% in the derivation and 23.2% in the validation cohort. Causal forest analysis revealed lactate level and Glasgow Coma Scale (GCS) score as key effect modifiers. A therapeutic target subgroup—defined as lactate ≥ 4.5 mmol/L and GCS ≤ 12—comprised 20.7% of the validation cohort. This subgroup showed a substantially greater mortality reduction with high-FFP transfusion (risk difference –13.3%, 95% CI –22.4 to –4.2%; number needed to treat [NNT] 7.5), compared with the overall cohort (risk difference –3.3%, 95% CI –6.7 to 0.5%; NNT 32.1). Results were consistent across sensitivity analyses.

**Conclusions:**

High FFP-to-RBC transfusion ratios may confer the greatest benefit in patients with impaired consciousness and metabolic acidosis. Identifying high-benefit subgroups using machine learning could support more individualized transfusion strategies in trauma care.

**Supplementary Information:**

The online version contains supplementary material available at 10.1186/s13054-025-05678-z.

## Introduction

Trauma remains one of the leading global health challenges, accounting for a significant burden of mortality and long-term disability. Trauma-induced coagulopathy represents a key complication of trauma, with transfusion strategies central to its management [1]. To effectively manage trauma-induced coagulopathy, it is necessary to follow a damage control resuscitation protocol, with an emphasis on stabilizing hemodynamic status and administering fresh frozen plasma (FFP) earlier than red blood cells (RBC) [2, 3].

Conventionally, an FFP-to-RBC ratio of 1 has been recommended [4], but recent studies have indicated that higher ratios may be effective for certain patient groups [5–8]. In previous research, we demonstrated that a high ratio of FFP to RBC transfusion (specifically, FFP-to-RBC ratio > 1) was associated with favorable outcomes in patients with severe blunt trauma [5]. However, trauma-induced coagulopathy in blunt trauma exhibits considerable heterogeneity, influenced by factors such as the degree of tissue hypoperfusion and the severity of injury [1]. In particular, traumatic brain injury is often accompanied by marked hyperfibrinolysis, further contributing to this heterogeneity [9, 10]. This complexity poses challenges in determining the optimal transfusion strategy across diverse patient presentations. These observations indicate the importance of developing more targeted approaches to identifying which patients are most likely to benefit from plasma-rich transfusion.

Recent advances in machine learning have led to novel approaches to evaluating heterogeneous treatment effects [11]. Algorithms such as causal forest can quantitatively elucidate the varying effects of treatments and have shown promise in clinical contexts characterized by high heterogeneity, such as trauma [12]. By analyzing large datasets, causal forest can identify distinct “high-benefit” subgroups among blunt trauma patients for whom high FFP transfusion may be more or less beneficial. This approach allows for a more nuanced understanding of treatment effects and the potential to tailor interventions to specific patient characteristics [13].

Given the complexity of coagulopathy in blunt trauma, we hypothesized that distinct subgroups exist in which high-FFP transfusion has varying effectiveness. This study aimed to evaluate heterogenous treatment effects of high-FFP transfusion in blunt trauma. We expected that identifying more effective treatment targets could meaningfully benefit clinical practice and contribute to improved patient outcomes.

## Methods

### Study design and setting

This study was a retrospective analysis of the Japan Trauma Data Bank (JTDB), a nationwide multicenter prospective trauma registry. This database collects data on trauma admissions from more than 200 participating hospitals in Japan and is compiled annually. Details of the JTDB are described in eAppendix 1 and elsewhere [5, 14]. All trauma patients with an Injury Severity Score (ISS) of at least 9 are to be registered by participating hospitals.

### Study participants

From all trauma cases registered in the JTDB between 2019 and 2023, we included adult patients with blunt trauma who sustained severe injuries (ISS ≥ 16) and required blood transfusion, consistent with previous findings [5]. An explanation of the ISS, as well as data on the exclusion criteria, are provided in eAppendix 2 and 3.

### Measurements, exposures and outcome

We collected the data indicated in eAppendix 4 from the database. We defined the intervention of interest as high-FFP transfusion (FFP-to-RBC ratio > 1) and the comparison as low-FFP transfusion (FFP-to-RBC ratio ≤ 1) according to FFP-to-RBC ratio within 24 h of hospital arrival. Primary outcome was all-cause in-hospital mortality. In our dataset, mortality had no missing values (eTable 1). Following previous studies that conducted heterogeneous treatment effect analyses using causal forests [13], we addressed covariate missingness using a non-parametric single imputation method (missForest). [15] Missing values were handled using the random forest approach to impute missing values, as detailed in eAppendix 4 and eTable 1. Blood transfusion products in Japan are explained in eAppendix 5 [15].

### Statistical methods

**Fig. 1:**
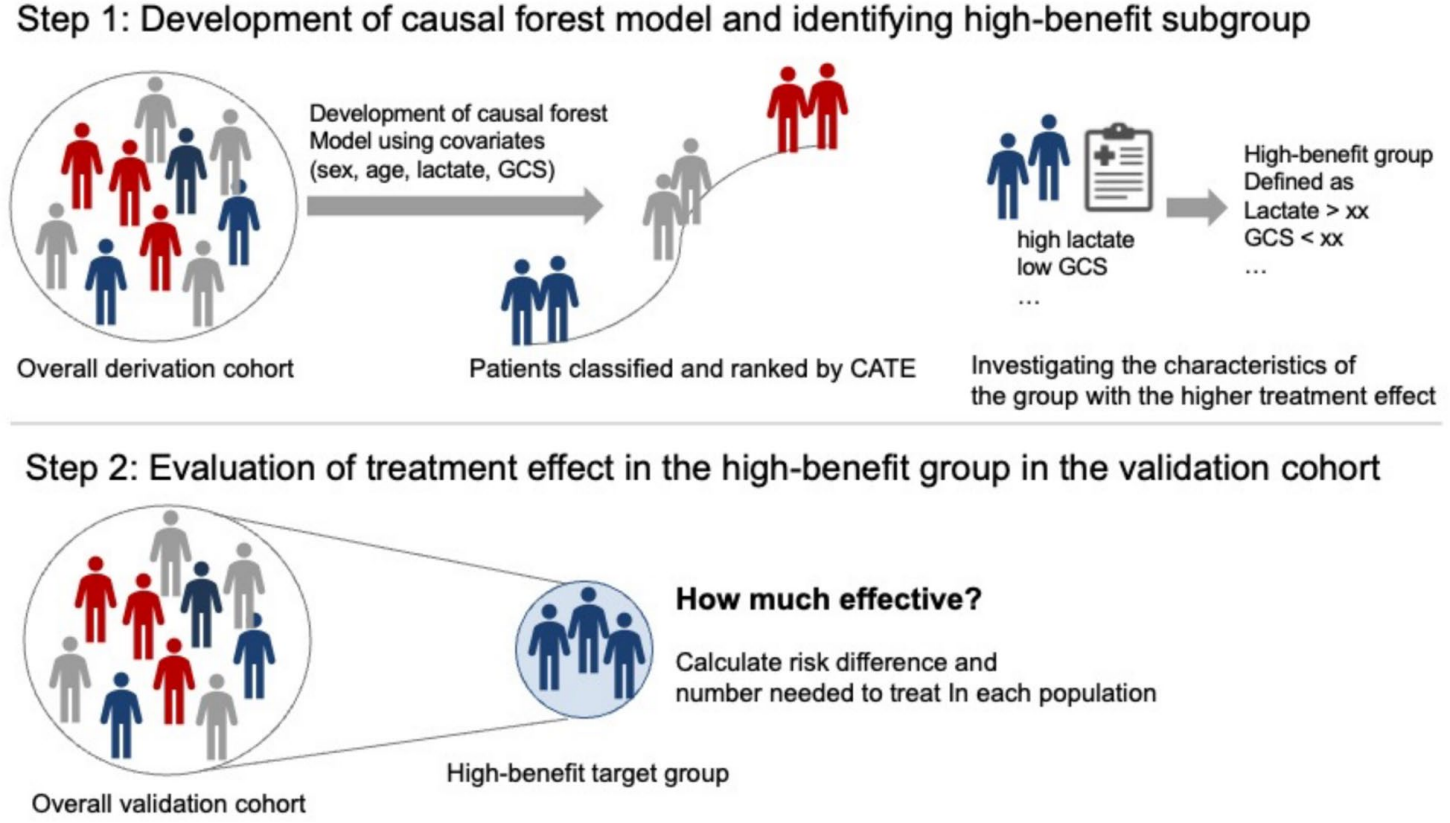
Analytic concept model of identifying and validating high-benefit subgroup using causal forest model. CATE: Conditional average treatment effect, GCS: Glasgow Coma Scale

The analytic concept model is described in Fig. 1. First, the study population was divided into a derivation cohort (patients from 2019 to 2021) used to develop the causal forest model and identify subgroups, and a validation cohort (patients from 2022 to 2023) used to confirm the validation of treatment effects. Patient characteristics in both derivation and validation cohorts were summarized by high- and low-FFP group. Continuous variables are reported as median with interquartile range (IQR), and categorical variables as counts and percentages.

To assess the heterogeneous treatment effects in the high-FFP group, we applied a machine learning–based causal forest model to the derivation cohort. This model estimates the conditional average treatment effect (CATE) and detects heterogeneity in high-dimensional settings. Our model was constructed using an honest structure, in which each tree was built by splitting the training data into two disjoint subsamples, one for learning the tree structure (splitting rules) and the second for estimating treatment effects [16]. We trained a total of 5,000 trees and we used default values of *grf* package for other hyperparameters of the model. Heterogeneous treatment effects and identification of high-benefit therapeutic targets were evaluated as described previously [16, 17].

A total of 18 covariates were included in the causal forest model, including age*, sex*, Charlson Comorbidity Index (CCI) [18]^*^, use of antithrombotic agents, mechanism of injury*, Glasgow Coma Scale (GCS)*, systolic blood pressure (SBP)*, heart rate (HR)*, respiratory rate (RR)*, body temperature (BT)*, lactate level (mmol/L)*, Abbreviated Injury Scale (body region 1 [head], 4 [thorax], 5 [abdominal and pelvic contents], and 8 [lower extremities and pelvis]), ISS*, and Trauma and Injury Severity Score (TRISS). A dummy-variable design matrix was used for model training.

After development of the causal forest model, CATE was estimated for each patient and used to stratify the population into five equal-sized quintile groups (Q1–Q5). To assess the calibration of the causal forest model, we compared the group-specific average treatment effect (ATE) estimated using augmented inverse probability weighting (AIPW) across the CATE-based quintile strata [16]. Model calibration was evaluated based on the best linear predictor using out-of-bag predictions. A coefficient closer to 1 was interpreted as indicating good calibration.

In the derivation cohort, we descriptively compared the characteristics across the five CATE quintile groups to assess potential effect modifiers [17]. We then selected the most influential and clinically plausible variables as final determinants of heterogeneity, based on both variable importance measures and clinical relevance. To further explore heterogeneity, we constructed 3 × 3 heatmaps by categorizing the key variables into tertiles [16]. We examined the mean CATE and inverse probability of treatment weighting (IPTW)-based risk differences in each of the nine subgroups to identify the therapeutic target. The variables marked above with an asterisk (*) were used to estimate the propensity score in the analysis of subgroups divided by heat maps and to estimate the propensity score in IPTW analysis in the subsequent validation cohort.

To evaluate the generalizability of the identified therapeutic target, we applied the same subgroup-defining criteria to the validation cohort. The treatment effect of high FFP-to-RBC ratio transfusion on in-hospital mortality was estimated as a risk difference with 95% confidence intervals, using a generalized linear model with an identity link, weighted by IPTW based on propensity scores. Hospital-level clustering was accounted for by specifying hospital identifiers in the model design, and robust (sandwich) standard errors were used to adjust for intracluster correlation. The number needed to treat (NNT) and its 95% confidence intervals were also calculated. Finally, treatment effects in the high-benefit therapeutic target were compared with those in the overall cohort to assess the added clinical utility of a targeted transfusion strategy [19].

A p-value < 0.05 was considered statistically significant. All analyses were performed using R (version 4.3.3).

### Sensitivity analysis

As a sensitivity analysis, we examined whether the treatment effect in the identified therapeutic target remained robust under different conditions:

1. We re-estimated the treatment effect of high FFP-to-RBC ratio transfusion in the therapeutic target after excluding patients who died within 120 min of arrival. This analysis aimed to mitigate the potential for survival bias, wherein patients who survive longer may be more likely to receive larger volumes of FFP [5].
2. We re-evaluated the treatment effect in an alternative validation cohort consisting of patients with blunt trauma who received transfusion within 2 h of hospital arrival, without applying the ISS ≥ 16 criterion. This inclusion criterion was intended to capture a broader range of patients with severe trauma requiring transfusion, not limited to those who met massive transfusion criteria. In practice, patients for whom transfusion is initiated within the first 2 h often reflect clinical recognition of significant injury severity, based on early physiological indicators and physician judgment in the acute phase of trauma care [20]. Although ISS ≥ 16 is often used to define severe trauma, its precise calculation is not feasible at the time of arrival. [21, 22] Therefore, this ISS-unrestricted cohort better captures the practical timing of transfusion initiation in acute care settings.
3. We evaluated the treatment effect using Cox proportional hazards models with IPTW and hospital-level clustering. Time from hospital arrival to death was used as the outcome, and hazard ratios (HRs) with 95% CI were estimated for the overall validation cohort and the predefined therapeutic target subgroup. In addition, Kaplan–Meier survival curves were plotted for visual comparison between groups.

## Results

### Patient characteristics and treatment details

**Fig. 2:**
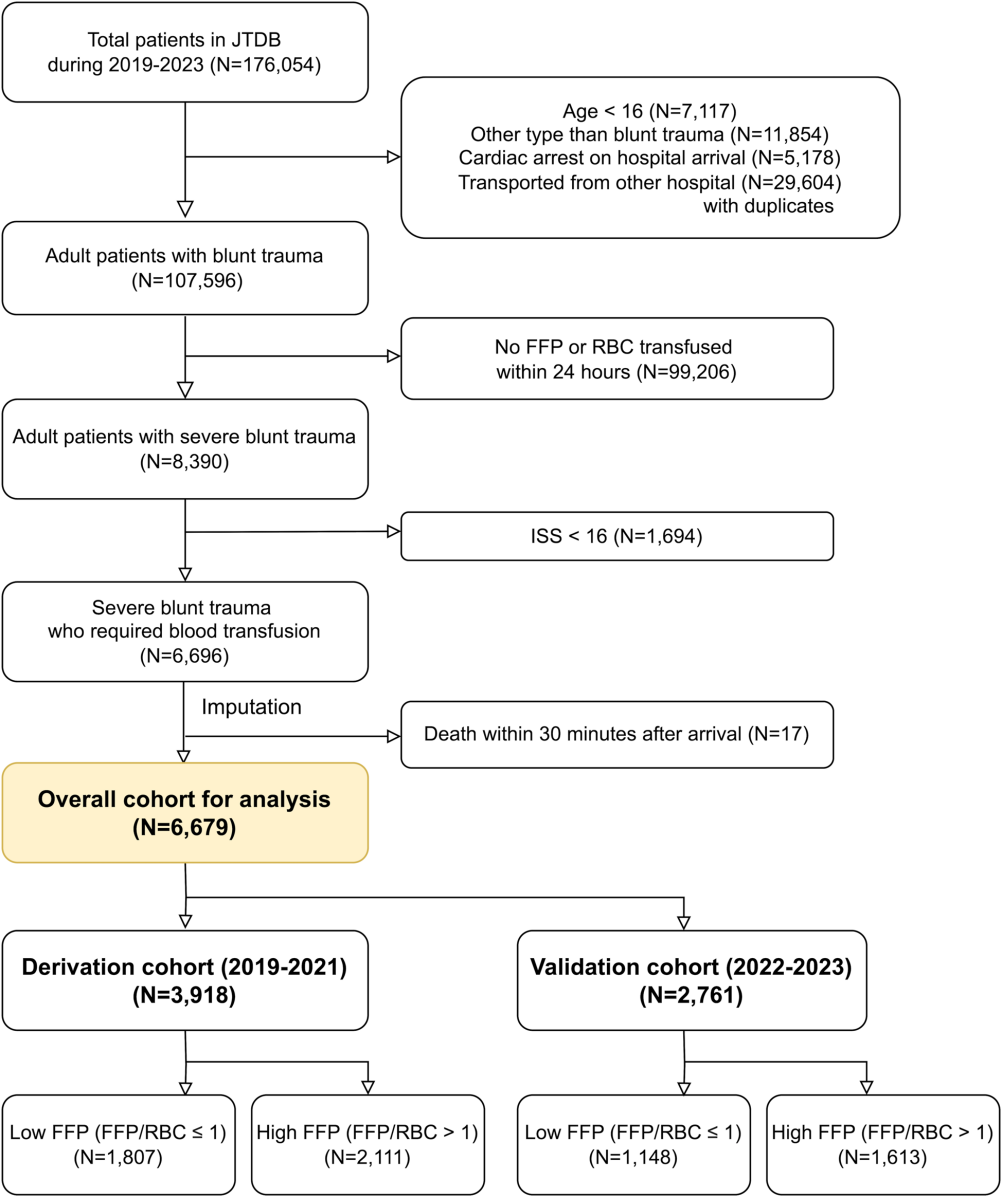
Study flowchart. JTDB: Japan Trauma Data Bank, AIS: Abbreviated Injury Scale, ISS: Injury Severity Score, FFP: Fresh frozen plasma, RBC: Red blood cell

A flowchart of the study is shown in Fig. 2. Finally, 6,679 patients from 205 hospitals were included in the analysis of the 176,054 patients enrolled in the JTDB.

In the derivation cohort, 2,111 (53.9%) received high-FFP transfusion. The cohort included 2,550 males (65.1%), and median age (interquartile range [IQR]) was 66 years (45–78). Median ISS was 26 (IQR, 22–34). The high-FFP group received a median of 14 units of FFP (IQR, 8–22) and 8 units of RBC (IQR, 4–14), whereas the low-FFP group received 6 units of FFP (IQR, 4–10) and 8 units of RBC (IQR, 4–14). In-hospital mortality was 912 (23.3%) overall, with 443 deaths (24.5%) in the low-FFP group and 469 deaths (22.2%) in the high-FFP group (eTable 2 and 3).

In the validation cohort, 1,613 (58.4%) received high-FFP transfusion. The cohort included 1,755 males (63.6%), median age 66 (45–79) and median ISS of 26 (22–34). The high-FFP group received 14 units of FFP (IQR, 8–22) and 6 units of RBC (IQR, 4–12), whereas the low-FFP group received 6 units of FFP (IQR, 4–10) and 8 units of RBC (IQR, 4–14). In-hospital mortality was 640 (23.2%) overall, with 271 deaths (23.6%) in the low-FFP group and 369 deaths (22.9%) in the high-FFP group (eTable 4 and 5).

### Development of the causal forest model in the derivation cohort

**Fig. 3:**
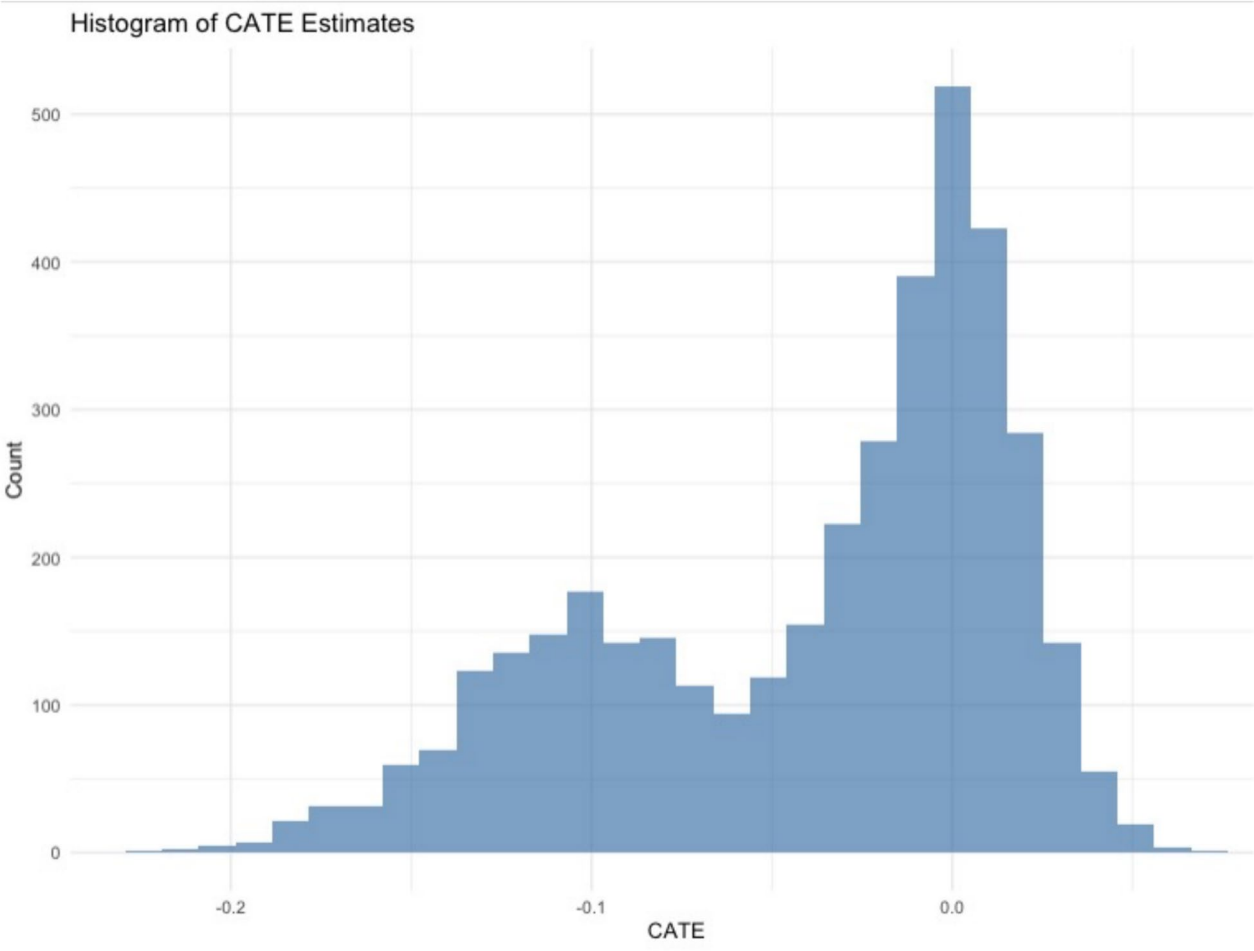
Distribution of CATE estimates from the causal forest model in the derivation cohort. The histogram shows a bimodal distribution of CATE estimates. Because the primary outcome was in-hospital mortality, negative CATE values suggest the presence of subgroups that could experience greater benefit from high FFP-to-RBC ratio transfusion. CATE, Conditional average treatment effect

In the derivation cohort, our causal forest model estimated the CATE for each individual, using in-hospital mortality as outcome. Distribution of the predicted CATEs exhibited a bimodal pattern, as shown in Fig. 3. Since mortality was the outcome, negative CATE values indicated a treatment benefit from high-FFP transfusion. Patients were stratified into quintiles based on the estimated CATEs and group-specific ATE were estimated using AIPW. The results were well-calibrated and plotted in eFigure 1.

### Variable selection for identifying the high-benefit therapeutic target

The characteristics of the groups divided by CATE quintiles are shown in Table 1. To identify a clinically meaningful therapeutic target based on the estimated CATEs, we first examined characteristics that were descriptively overrepresented in the lowest-CATE quintile (Q1), which represented the group with the highest estimated treatment benefit. Four physiologically relevant and clinically observable variables were identified as potential effect modifiers —lactate level, GCS, SBP, and BT. We also assessed the variable importance of these features in the causal forest model (eFigure 2). Considering both statistical contribution and clinical practicality, and following discussion between two experts (GF and YO), we selected lactate and GCS as the final effect modifiers for subgroup stratification. To ensure robustness, we also conducted the same analyses using SBP and BT—variables excluded in the selection process—as potential effect modifiers (eAppendix 6, eFigure 3, and eTable 6). While several candidate subgroups incorporating SBP or BT were evaluated, the subgroup defined by lactate and GCS consistently showed the greatest estimated treatment benefit and clinical interpretability. These findings further support the appropriateness of our final variable selection.

**Table 1:** Patient characteristics by CATE quintile

Characteristic	Q1 (Most benefited group)	Q2	Q3	Q4	Q5 (Least benefited group)
Total (n)	784	783	784	783	784
Male, n (%)	509 (64.9%)	503 (64.2%)	537 (68.5%)	518 (66.2%)	483 (61.6%)
Age, median [IQR]	71 [56–80]	67 [47–78]	51 [34–74]	54 [36–76]	72 [59–79]
CCI, median [IQR]	0 [0–1]	0 [0–0]	0 [0–0]	0 [0–0]	0 [0–1]
Antithrombotic medication, n (%)	71 (9.1%)	50 (6.4%)	45 (5.7%)	33 (4.2%)	95 (12.1%)
Mechanism of injury, n(%)					
Pedestrian, n (%)	189 (24.1%)	146 (18.6%)	115 (14.7%)	115 (14.7%)	145 (18.5%)
Bicycle, n (%)	62 (7.9%)	100 (12.8%)	49 (6.2%)	51 (6.5%)	65 (8.3%)
Motorbike, n (%)	85 (10.8%)	85 (10.9%)	138 (17.6%)	135 (17.2%)	71 (9.1%)
Car, n (%)	93 (11.9%)	89 (11.4%)	127 (16.2%)	143 (18.3%)	113 (14.4%)
Fall, n (%)	255 (32.5%)	254 (32.4%)	256 (32.7%)	236 (30.1%)	252 (32.1%)
Slip, n (%)	51 (6.5%)	49 (6.3%)	36 (4.6%)	37 (4.7%)	64 (8.2%)
Others, n (%)	49 (6.2%)	60 (7.7%)	63 (8%)	66 (8.4%)	74 (9.4%)
HR (bpm), median [IQR]	102 [81–122]	102 [81–124]	102 [81–124]	92 [79–109]	89 [76–102]
SBP (mmHg), median [IQR]	96 [69–141]	115 [79–150]	113 [90–140]	118 [96–139]	119 [97–146]
RR (/minutes), median [IQR]	25 [19–30]	22 [18 –28]	24 [20–30]	23 [19–27]	21 [18–25]
BT (℃), median [IQR]	35.8 [35.1–36.3]	36.1 [35.7–36.5]	36.2 [35.8–36.6]	36.3 [35.9–36.7]	36.3 [35.9–36.6]
Lactate (mmol/L), median [IQR]	4.9 [3.0–7.6]	4.1 [2.6–6.3]	4.1 [2.7–5.6]	3.0 [2.1–3.9]	2.5 [1.8–3.5]
GCS, median [IQR]	6 [3–10]	6 [3–12]	14 [11–15]	14 [13–15]	14 [12–15]
AIS, median [IQR]					
Body region 1 (Head)	5 [1–6]	5 [3–6]	3 [1–5]	1 [1–4]	1 [1–4]
Body region 4 (Thorax)	4 [1–5]	4 [1–4]	4 [1–4]	4 [1–4]	3 [1–4]
Body region 5 (Abdomen)	1 [1–4]	1 [1–3]	1 [1–4]	1 [1–4]	1 [1–3]
Body region 8 (Lower Extremities)	3 [1–5]	3 [1–5]	3 [1–4]	3 [1–4]	3 [1–4]
ISS, median [IQR]	34 [26–43]	34 [25–41]	26 [22–33]	24 [18 –29]	22 [18–26]
TRISS, median [IQR]	0.348 [0.200–0.494]	0.530 [0.227 - 0.672]	0.887 [0.756–0.946]	0.922 [0.849 - 0.962]	0.887 [0.801–0.934]

Patients in the derivation cohort were stratified into five quintile groups (Q1–Q5) based on the estimated conditional average treatment effect (CATE) from the causal forest model. A lower CATE value indicates a greater treatment benefit from high-FFP transfusion. Therefore, Q1 represents the group with the highest estimated benefit, and Q5 the lowest. IQR: Interquartile range, CCI: Charlson Comorbidity Index, HR: Heart rate, SBP: Systolic blood pressure, RR: Respiratory rate, BT: Body temperature, GCS: Glasgow Coma Scale, AIS: Abbreviated Injury Scale, ISS: Injury Severity Score, TRISS: Trauma and Injury Severity Score.

### Identification of high-benefit therapeutic target using heatmaps

**Fig. 4:**
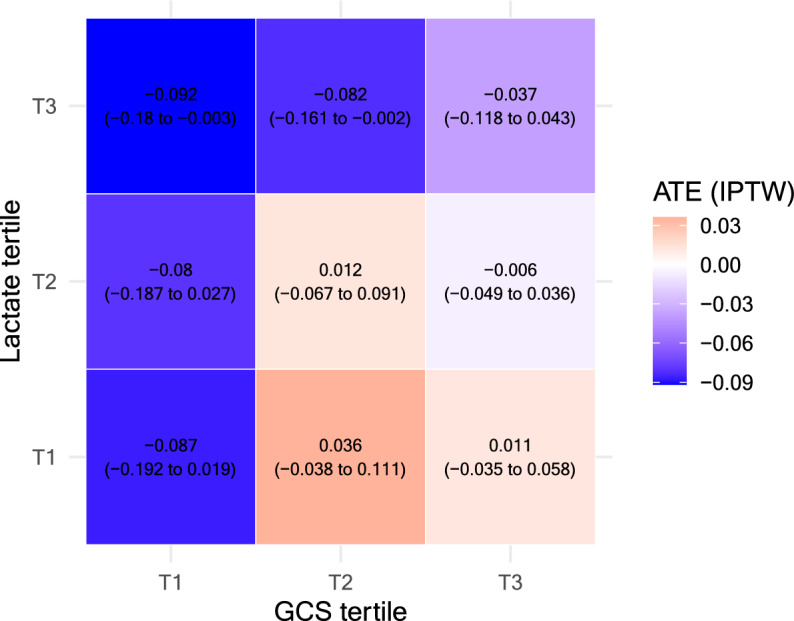
Subgroup heatmaps of treatment effect heterogeneity based on causal forest analysis. Heatmap of subgroup ATE estimated using IPTW. Each cell shows the risk difference and corresponding 95% confidence interval for high versus low FFP transfusion within the defined subgroup. Darker blue colors indicate greater estimated mortality reduction associated with high FFP transfusion. Lactate tertile: T1 (- 2.65), T2 (2.66—4.59), T3 (4.60 -). GCS tertile: T1 (3—7), T2 (8—12), T3 (14—15). ATE: Average treatment effect, CATE: Conditional average treatment effect, FFP: Fresh frozen plasma, GCS: Glasgow Coma Scale, IPTW: Inverse probability treatment weighting

To explore the interaction between these two selected variables and treatment effect heterogeneity, we constructed 3 × 3 heatmaps by dividing GCS and lactate levels into tertiles. The resulting nine subgroups were evaluated for group-specific ATE, represented as estimated risk differences calculated using the weighted method by IPTW (Fig. 4). For reference, the corresponding heatmap based on mean CATE values is presented in eFigure 4. Based on the heatmap, we heuristically identified a candidate therapeutic target characterized by patients in the highest lactate tertile (T3) and lowest or middle GCS tertiles (T1 and T2), namely with lactate levels ≥ 4.6 mmol/L and GCS scores ≤ 13. Although this subgroup was initially derived from tertile-based cutoffs on the heatmap, we further refined them by approximating to commonly used clinical thresholds [23]. This adjustment was made to enhance clinical interpretability and usability in practice. The final target definitions were as below, and the characteristics of this group are shown in eTable 7.

• **Therapeutic Target**: Lactate ≥ 4.5 mmol/L and GCS ≤ 12.

### Treatment effect of each subgroup in the validation cohort

In the overall validation cohort, the estimated risk difference in in-hospital mortality for patients receiving high FFP-to-RBC ratio transfusion compared to low ratio transfusion was –3.3% (95% CI: −7.0 to 0.3%), with an NNT of 32.1 (95% CI: 14.6 to 244.2). In the high-benefit therapeutic target (lactate ≥ 4.5 mmol/L and GCS ≤ 12), the risk difference was –13.3% (95% CI: −22.4 to −4.2%), with an NNT of 7.5 (95% CI: 4.5 to 23.8). These results are summarized in Table 2, along with the results of the three sensitivity analyses. In sensitivity analysis 2, which used an ISS-unrestricted validation cohort defined by transfusion within 2 h of hospital arrival, a total of 1,804 patients were included, of whom 993 (55.0%) received high-FFP transfusion. The flowchart of patient selection is shown in eFigure 5. Kaplan–Meier survival curves for sensitivity analysis 3 are presented in eFigure 6. The results of these sensitivity analyses were consistent with those of the primary analysis, supporting the robustness of the predefined therapeutic target.

**Table 2:** Treatment effect in the overall cohosrt and the derived therapeutic target

Main analysis	RD (95% CI) of high FFP group	NNT
Overall cohort (N = 2,761)	−3.3% (−7.0 to 0.3%)	29.9 (14.2 to 287.5)
Therapeutic Target (N = 571)		
Lactate ≥ 4.5 and GCS ≤ 12	−13.3% (−22.4 to −4.2%)	7.5 (4.5 to 23.8)
Sensitivity analysis 1 (exclude death within 120 min of hospital arrival)		
Overall cohort (N = 2,742)	−2.9% (−6.5 to 0.7%)	34.5 (15.5 to 147.2)
Therapeutic Target (N = 557)		
Lactate ≥ 4.5 and GCS ≤ 12	−12.7% (−21.9 to −3.5%)	7.9 (4.6 to 28.4)
Sensitivity analysis 2 (ISS-unrestricted cohort: transfusion initiated within 2 h of arrival)		
Overall cohort (N = 1,804)	−4.8% (−9.8 to 0.2%)	20.8 (10.2 to 499.0)
Therapeutic Target (N = 464)		
Lactate ≥ 4.5 and GCS ≤ 12	−11.2% (−21.2 to −1.2%)	8.9 (4.7 to 85.6)
Sensitivity analysis 3 (Survival analysis using Cox proportional hazards model)		
Overall cohort (N = 2,709)	0.84 (0.70–1.01)	N/A
Therapeutic Target (N = 559)		
Lactate ≥ 4.5 and GCS ≤ 12	0.64 (0.49–0.85)	N/A

CI: Confidence interval, GCS: Glasgow Coma Scale, ISS: Injury severity scale, NNT: Number needed to treat, RD: Risk difference

## Discussion

### Key observation

In this multicenter cohort study of patients with severe blunt trauma requiring transfusion, we applied a machine learning–based causal forest model and observed heterogeneous treatment effects of high FFP-to-RBC transfusion ratios. We identified a therapeutic target—defined by GCS ≤ 12 and lactate ≥ 4.5 mmol/L—with a substantially greater reduction in mortality. These findings suggest that tailoring transfusion strategies using readily available clinical indicators may improve outcomes.

### Strength and clinical implications

A key strength of this study is the use of causal forest modeling to explore heterogeneity in treatment effects beyond average-based analyses. Unlike traditional subgroup methods, this machine learning approach identifies patient-level variability without requiring predefined strata, offering novel insights into individualized transfusion practices. [13, 24]

Importantly, our model translated complex output into actionable thresholds—lactate ≥ 4.5 mmol/L and GCS ≤ 12—that are readily applicable in clinical settings. This enables targeted plasma-based resuscitation, potentially improving outcomes and optimizing resource use.

These findings also inform future trial design. Rather than enrolling unselected trauma populations, studies may be more effective by targeting patients who are more likely to benefit from early interventions, such as those with early signs of hypoperfusion or neurologic compromise. This precision-based strategy could enhance both the efficiency and clinical relevance of trauma resuscitation research.

Another strength of our study is that it was conducted in Japan, where awareness of balanced transfusion has been high. The Japanese trauma guidelines recommend an approximate 1:1 ratio of FFP to RBC in massive transfusion, and a nationwide study has reported a temporal increase in the use of higher FFP-to-RBC ratios in tertiary emergency hospitals [25]. This environment enabled us to investigate treatment effects in a relatively high-FFP population, providing insights that may be informative for trauma systems internationally.

### Interpretation

The benefit of high FFP transfusion observed in this study may be explained by two key physiological indicators: elevated lactate levels and impaired consciousness. These reflect distinct mechanisms of trauma-induced coagulopathy and help identify patients most likely to benefit from plasma-rich transfusion.

Elevated lactate levels indicate systemic hypoperfusion and shock—major triggers of coagulopathy through endothelial activation, inflammation, and glycocalyx degradation. [26–28] This results in impaired coagulation and increased bleeding risk. Plasma may counteract this cycle by replenishing clotting factors and stabilizing the endothelium, restoring hemostasis. [26, 28]

Impaired consciousness likely reflects traumatic brain injury (TBI), a condition characterized by tissue factor release and hyperfibrinolysis [9, 10, 29], and this early-onset coagulopathy may require plasma-based resuscitation. Studies have shown limited benefit of whole blood transfusion in TBI [30] and suggest that plasma-rich strategies may be more effective than balanced transfusion protocols. [31, 32] This interpretation aligns with findings from a secondary analysis of the PAMPer trial, in which prehospital plasma administration was associated with improved survival in TBI patients—particularly those with severe neurologic impairment (GCS < 8) or concomitant polytrauma [33]. This may relate to the need for early correction of specific coagulation deficits.

These interpretations are supported by recent proteomic evidence. In one study, plasma transfusion provided more favorable modulation of coagulation and immune-related proteins than low-titer group O whole blood, particularly in patients with TBI or severe shock. [34] These molecular changes may help explain the improved outcomes seen in our high-benefit therapeutic target defined by elevated lactate and impaired consciousness.

In summary, patients with both lactate elevation and impaired consciousness may represent a physiological state in which coagulopathy progresses rapidly and standard transfusion is insufficient. In such cases, early plasma-focused resuscitation may better support coagulation and vascular stability, improving outcomes.

### Limitations

This study has several limitations. First, as it was an observational analysis based on registry data, causal effects cannot be directly inferred. Although we may consider that strong unmeasured confounding that would substantially bias the results is unlikely, there remains a potential for time-dependent unmeasured confounding—for example, patients who survive longer may have a greater chance of receiving more FFP. To address this issue, we conducted validation using a temporally distinct cohort and performed multiple sensitivity analyses, which yielded results consistent with the primary analysis and supported its robustness. Second, we included patients with an ISS of ≥ 16 and those who received transfusion to target a population with clinically significant trauma. ISS is validated but not available at arrival, limiting real-time use. In real-world settings, the initiation of transfusion is largely based on physician judgment, informed by the mechanism of injury, vital signs, and other early indicators of severity. To partly address this limitation, we conducted a sensitivity analysis using an ISS-unrestricted cohort defined by transfusion initiation within 2 h of arrival. This approach may better reflect actual triage decisions in emergency care. Third, as this study was conducted using data from a Japanese nationwide trauma registry, the generalizability of the findings to other countries or healthcare systems remains uncertain. In particular, trauma epidemiology in Japan is characterized by a markedly older patient population, reflecting the country’s advanced demographic aging. This pattern is influenced by national-level demographic trends as well as several injury-related factors, including a low incidence of violence-related trauma and a very high helmet-use compliance rate among motorbike riders, which reduces high-energy head injuries in younger populations. While this older age distribution may limit the applicability of our findings to regions with younger trauma populations, the global trend toward population aging suggests that our results may become increasingly relevant to other countries in the future. Furthermore, differences in transfusion practice—such as the relatively limited use of platelet transfusion in Japan—may also influence the extent to which these findings can be extrapolated to settings with different resource availability or clinical protocols. Finally, the registry used in this study did not contain data on coagulation or fibrinolysis markers, such as thromboelastography or conventional laboratory coagulation tests. The inclusion of such parameters could have provided deeper understanding of the underlying pathophysiology of trauma-induced coagulopathy and further elucidated the mechanisms by which high FFP transfusion improves outcomes.

## Conclusion

In patients with severe blunt trauma requiring transfusion, we identified heterogeneous treatment effects of high FFP-to-RBC ratio transfusion. A high-benefit therapeutic target (GCS ≤ 12, lactate ≥ 4.5 mmol/L) showed a substantially greater survival benefit. These findings support the clinical utility of implementing targeted transfusion strategies guided by readily available physiological indicators in trauma care, which should be further evaluated in randomized controlled trials.

## Supplementary Information

Supplementary materials 1

## Data Availability

A general overview of the Japan Trauma Data Bank can be found at http://www.jtcr-jatec.org/traumabank/index.htm. The data used for the current analysis were obtained from Japan Trauma Care and Research. Because access to the JTDB is regulated, the datasets supporting this study are not publicly available.
