# Supplementary material for: Therapeutic target of high fresh frozen plasma to red blood cell ratio in severe blunt trauma
Source: Crit Care. 2025 Oct 16;29:436. doi: 10.1186/s13054-025-05678-z (PMC12532888; doi:10.1186/s13054-025-05678-z)
Supplement: Supplementary file 1 — Supplementary materials 1 [file 13054_2025_5678_MOESM1_ESM.docx]

**Supplementary File**

**Contents**

**eAppendix 1. Explanation of Japan Trauma Data Bank**

**eAppendix 2. Explanation of AIS and ISS**

**eAppendix 3. The details of exclusion criteria and missing data management**

**eAppendix 4. Analyses using SBP and BT as candidate effect modifiers**

**eTable 1. Proportion of missing value of each variable**

**eTable 2. Patient characteristics in derivation cohort (2019-2021)**

**eTable 3. Treatment details, adverse events, and outcome in derivation cohort (2019-2021)**

**eTable 4. Patient characteristics in validation cohort (2022-2023)**

**eTable 5. Treatment details, adverse events, and outcome in validation cohort (2022-2023)**

**eTable 6. Treatment effect in each derived therapeutic target**

**eTable 7. Patient characteristics in the target subgroup (GCS ≤12 and lactate ≥4.5 mmol/L) in the validation cohort**

**eFigure 1. Group-specific ATE estimates across CATE quintiles using AIPW and OLS estimators.**

**eFigure 2. Variable importance in the causal forest model.**

**eFigure 3. Exploratory heatmaps of treatment effect using lactate level, GCS, SBP, and BT.**

**eFigure 4. Subgroup heatmaps of treatment effect using lactate level and GCS.**

**eFigure 5. Flowchart of patient selection for the validation cohort used in sensitivity analyses.**

**eFigure 6. Kaplan–Meier survival curves of patients in the overall and therapeutic target population of the validation cohort.**

**eAppendix 1. Explanation of Japan Trauma Data Bank**

The data were obtained from the JTDB dataset, which contains information from 303 hospitals across Japan as of 2022. The JTDB is a nationwide, multicenter, prospective, observational trauma registry established in 2003 by the Trauma Registry Committee of the Japanese Association for the Surgery of Trauma and the Committee for Clinical Care Evaluation of the Japanese Association for Acute Care Medicine, and managed by the Japan Trauma Care and Research (JTCR), which is a nonprofit organization for trauma research. This registry was developed to improve the quality of trauma care through the collection of prehospital information, clinical information during the hospital stay, data on trauma diagnoses based on the Abbreviated Injury Scale, and Injury Severity Score, and data on mortality-related outcomes. These data are compiled by administrators based on the in-hospital charts and prehospital records, which are routinely submitted by paramedics to the hospitals. Nearly all the institutions participating in the JTDB are government-certified tertiary emergency and critical care centers. Details on the JTDB have been published previously. Anonymized data from the JTDB are available to affiliated researchers of the JTCR, and we obtained these data from the JTCR.

This registry was modified in 2019 and the data items to be collected were changed; data including the amount of blood transfusion within 24 hours became a new entry item after the new system was introduced. In this study, we used the JTDB 2019 registry cases after the introduction of the new system.

Total of 288 hospitals were registered in JTDB database. Information of registered institutions and summary of JTDB 2019 are available at on JTDB website by JTCR. (https://www.jtcr-jatec.org/traumabank/dataroom/data/JTDB2020e.pdf)

**eAppendix 2. Explanation of AIS and ISS**

The Abbreviated Injury Scale (AIS) is anatomically based, globally accepted severity scoring system used to classify individual injuries by body region. AIS code consists of total 7 digits code; 6 digits code for the detail of injury and last 1 digit code for severity score. First 1 digit means body region (eg, head, neck), second 3 digits mean anatomical structure, and third 2 digits mean the level of injury. If the AIS code is written as 123456.7, it means 1: Body region, 2, 3, 4: Anatomical structure, 5,6: Level of injury, 7: Severity score.

Ex. AIS: 140652.4

1: Head

406: Cerebrum

52: Thin subdural hematoma; hemorrhage of 50ml or less

4: Severe injury

**Injury Severity Scale (ISS)**

ISS is a severity scale calculated from AIS to assess the multiply injured patient. For the calculation, identify the highest three AIS score in each following six areas of the body. The score ranges from 1 to 75. If the AIS severity of any region of injury is 6 the score is 75.

1: Head and Neck

2: Face

3: Chest and thoracic spine

4: Abdomen, Lumber spine and Pelvic contents

5: Bony pelvis and limbs

6: Body surface

Add together the squares of the highest scores in three different body areas.

Ex.

Head and Neck: AIS severity 4, Chest: AIS severity 3, Abdomen: AIS severity 2, and Body surface: AIS severity 1,

ISS=4^2^+3^2^+2^2^=16+9+4=29 (The three highest score are included in the calculation.)

**eAppendix 3. The details of exclusion criteria**

**Exclusion Criteria**

The exclusion criteria were as follows: (a) younger than 16 years of age; (b) cardiac arrest (predefined as a heart rate of 0 or systolic blood pressure of 0 or respiratory rate] of 0) on arrival to the hospital; (c) major causes of injury were burns or any penetrating or unknown injuries; and (d) transferred from other hospitals, and then additionally (e) Injury Severity Score (ISS) score less than 16 and (f) did not receive RBC or FFP transfusion. After imputing missing data in the resulting dataset (details of missing variables and imputation are described in eTable 1), (g) cases that died within 30 minutes of arrival were excluded, and the final analysis was conducted.

**eAppendix 4. Data collection and data management**

**Data collection and Data management**

Missing data were imputed by the random forest method for the study cohort using the missForest package.[1] Random Forest imputation is a nonparametric algorithm that addresses nonlinearities and interactions and does not require a specific parametric model.[2] Using this method, single-point estimates are generated by random draws from independent normal distributions centered on the conditional mean predicted by the random forest, which uses bootstrap aggregation of multiple regression trees to reduce the risk of overfitting and combining estimates from many trees. Missing measures were imputed using following variables: sex, age, admission year, Charlson Comorbidity Index (CCI)[3], history of daily antithrombotic medication, mechanism of injury (e.g., bicycle, pedestrian, fall), prehospital vital signs (systolic blood pressure [SBP], diastolic blood pressure [DBP], heart rate [HR], respiratory rate [RR], and body temperature [BT]), transport time, vital signs (SBP, DBP, HR, RR, and BT) on arrival at the hospital, Glasgow Coma Scale (GCS) on arrival at the hospital, lactate (mmol/L) on arrival at the hospital, AIS (0 to 6) of each part, ISS, amount of blood products (RBC, FFP, Platelet concentrate [PC]) transfused within 24 hours of arrival at the hospital, use of tranexamic acid, procedure of any surgery, and damage control surgery, length of hospital stay, Glasgow Outcome Scale at discharge, and in-hospital mortality. Lactate levels were recorded in either mg/dL or mmol/L depending on the testing equipment used at each facility. Values expressed in mg/dL were converted to mmol/L by multiplying by 0.111.

**eAppendix 5. Explanation of blood products in Japan**

**Explanation of blood products in Japan**

In Japan, blood products, such as RBC and FFP, which are used in medical institutions, are supplied by the Japanese Red Cross Society (Tokyo, Japan). Two units of RBC (one unit: 140 mL, two units: 280 mL) and FFP (one unit: 120 mL, two units: 240 mL) were used in a single pack.

**eAppendix 6. Analyses using SBP and BT as candidate effect modifiers**

**Identification of candidate therapeutic subgroups using SBP or BT**

To assess whether systolic blood pressure (SBP) or body temperature (BT) could serve as meaningful effect modifiers for treatment heterogeneity, we constructed additional 3×3 heatmaps by dividing candidate variable pairs into tertiles. Specifically, SBP and BT, as well as BT in combination with GCS or lactate, were explored in an analogous manner to the primary analysis. The resulting subgroups were evaluated for their CATE and group-level ATE, expressed as risk differences estimated using IPTW. The full heatmap results are shown in eFigure 4.

Based on the group-level ATEs, we heuristically identified five candidate therapeutic subgroups as follows:

- Target 1: Lactate ≥ 2.66 mmol/L and BT ≤ 35.9°C
- Target 2: SBP ≤ 131 mmHg and BT ≤ 35.9°C
- Target 3: GCS ≤ 7 and BT ≤ 35.9°C
- Target 4: Lactate ≥ 4.60 mmol/L and SBP ≤ 131 mmHg
- Target 5: GCS ≤ 7 and SBP ≤ 131 mmHg

**Treatment effect of each subgroup in the validation cohort**

Each candidate subgroup was subsequently extracted from the validation cohort to estimate the risk difference in in-hospital mortality associated with high versus low FFP-to-RBC transfusion ratios. Point estimates and 95% confidence intervals were calculated using IPTW-weighted risk differences, and the full results are presented in eTable 6. Among these, Target 4 (Lactate ≥ 4.60 mmol/L and SBP ≤ 131 mmHg) and 5 (GCS ≤ 7 and SBP ≤ 131 mmHg) were the subgroups that showed a potentially meaningful treatment effect. However, these group were relatively small, and although the point estimate favored benefit, the magnitude were modest and the confidence interval wide. These limitations reduce their practical prioritization compared with the primary subgroup identified in the main analysis.

**eTable 1. Proportion of missing value of each variable**

| **Parameters** | **Cohort of adult patients with severe blunt trauma who required transfusion** |
| --- | --- |
|  | **N=6696** |
| **GOS at discharge** | 31.5% |
| **Prehospital DBP** | 21.1% |
| **Prehospital SBP** | 15.5% |
| **Lactate** | 14.8% |
| **BT at the hospital** | 11.0% |
| **Prehospital RR** | 9.6% |
| **Prehospital HR** | 6.2% |
| **DBP at the hospital** | 5.0% |
| **GCS at the hospital** | 1.3% |
| **TRISS** | 1.3% |
| **Sex** | 0.4% |

**HR: Heart rate, SBP: Systolic blood pressure, RR: Respiratory rate, BT: Body temperature, GCS: Glasgow coma scale, GOS: Glasgow Outcome Scale, TRISS: Trauma and Injury Severity Score.**

**eTable 2. Patient characteristics in derivation cohort (2019-2021)**

| **Characteristics** | **Total** | **Low FFP group** | **High FFP group** |
| --- | --- | --- | --- |
|  | **N=3918** | **N=1807** | **N=2111** |
| **Sex (male), n, (%)** | 2550 (65.1%) | 1149 (63.6%) | 1401 (66.4%) |
| **Age(years), median, (IQR)** | 66 [45 - 78] | 69 [48 - 79] | 63 [42 - 77] |
| **CCI, median, (IQR)** | 0 [0 - 1] | 0 [0 - 1] | 0 [0 - 0.5] |
| **Antithrombotic medication, n, (%)** | 294 (7.5%) | 141 (7.8%) | 153 (7.2%) |
| **Mechanism of injury, n, (%)** |  |  |  |
| **Pedestrian (traffic accident)** | 710 (18.1%) | 333 (18.4%) | 377 (17.9%) |
| **Bicycle (traffic accident)** | 327 (8.3%) | 149 (8.2%) | 178 (8.4%) |
| **Motorbike (traffic accident)** | 514 (13.1%) | 227 (12.6%) | 287 (13.6%) |
| **Car (traffic accident)** | 565 (14.4%) | 259 (14.3%) | 306 (14.5%) |
| **Slip** | 237 (6%) | 132 (7.3%) | 105 (5%) |
| **Fall** | 1253 (32%) | 548 (30.3%) | 705 (33.4%) |
| **Others** | 312 (8%) | 159 (8.8%) | 153 (7.2%) |
| **Vital signs, median, (IQR)** |  |  |  |
| **HR (bpm)** | 96 [79 - 116] | 95 [77 - 114] | 98 [80 - 120] |
| **SBP (mmHg)** | 113 [86 - 143] | 112 [85 - 142] | 114 [87 - 144 |
| **RR (/minutes)** | 23 [19 - 29] | 23 [19 - 28] | 23 [19 - 29] |
| **BT (℃)** | 36.1 [35.7 - 36.6] | 36.1 [35.7 - 36.5] | 36.2 [35.7 - 36.6] |
| **GCS, median, (IQR)** | 13 [6 - 14] | 13 [7 - 14] | 12 [6 - 14] |
| **Lactate (mmol/L), median, (IQR)** | 3.4 [2.3 - 5.4] | 3.3 [2.2 - 5.3] | 3.5 [2.4 - 5.5] |
| **AIS, median, (IQR)** |  |  |  |
| Body region 1 (head) | 3 [1 - 5] | 3 [1 - 5] | 4 [1 - 6] |
| Body region 4 (thorax) | 4 [1 - 4] | 4 [1 - 4] | 4 [1 - 4] |
| Body region 5 (abdomen) | 1 [1 - 3] | 1 [1 - 3] | 1 [1 - 3] |
| Body region 8 (lower extremity) | 3 [1 - 4] | 3 [1 - 4] | 3 [1 - 4] |
| **ISS, median, (IQR)** | 26 [22 - 34] | 25 [21 - 34] | 29 [22 - 36] |
| **TRISS, median, (IQR)** | 0.79 [0.49 - 0.92] | 0.81 [0.54 - 0.92] | 0.78 [0.48 - 0.92] |

**IQR: Interquartile range, HR: Heart rate, SBP: Systolic blood pressure, RR: Respiratory rate, BT: Body temperature, GCS: Glasgow Coma Scale, AIS: Abbreviated Injury Scale, ISS: Injury Severity Score, TRISS: Trauma and Injury Severity Score.**

**eTable 3. Treatment details, adverse events, and outcome in derivation cohort (2019-2021)**

| **Parameters** | **Total** | **Low FFP group** | **High FFP group** |
| --- | --- | --- | --- |
|  | **N=3918** | **N=1807** | **N=2111** |
| **Transfusion within 24 hours (units), median, (IQR)** |  |  |  |
| RBC | 8 [4 - 14] | 8 [4 - 14] | 8 [4 - 14] |
| FFP | 10 [6 - 18] | 6 [4 - 10] | 14 [8 - 22] |
| PC | 0 [0 - 10] | 0 [0 - 10] | 0 [0 - 10] |
| **FFP-to-RBC ratio, median, (IQR)** | 1.2 [1 - 2] | 1.0 [0.7 - 1.0] | 1.8 [1.4 - 2.3] |
| **Tranexamic acid, n, (%)** | 1925 (49.1%) | 821 (45.4%) | 1104 (52.3%) |
| **Surgical intervention, n, (%)** |  |  |  |
| Overall surgical procedure | 3097 (79%) | 1394 (77.1%) | 1703 (80.7%) |
| Damage control surgery | 646 (16.5%) | 297 (16.4%) | 349 (16.5%) |
| **Adverse event, n, (%)** |  |  |  |
| Overall adverse event | 1402 (35.8%) | 600 (33.2%) | 802 (38%) |
| Pulmonary edema | 24 (0.6%) | 10 (0.6%) | 14 (0.7%) |
| Pulmonary thromboembolism | 46 (1.2%) | 25 (1.4%) | 21 (1%) |
| Acute respiratory distress syndrome | 34 (0.9%) | 12 (0.7%) | 22 (1%) |
| Pneumonia | 465 (11.9%) | 203 (11.2%) | 262 (12.4%) |
| Acute Kidney Injury | 101 (1.7%) | 39 (1.5%) | 62 (1.8%) |
| Sepsis | 101 (2.6%) | 39 (2.2%) | 62 (2.9%) |
| **In-hospital mortality, n, (%)** | 912 (23.3%) | 443 (24.5%) | 469 (22.2%) |

**IQR: Interquartile range, RBC: Red blood cell, FFP: Fresh frozen plasma, PC: Platelet concentrate.**

**eTable 4. Patient characteristics in validation cohort (2022-2023)**

| **Characteristics** | **Total** | **Low FFP group** | **High FFP group** |
| --- | --- | --- | --- |
|  | **N=2761** | **N=1148** | **N=1613** |
| **Sex (male), n, (%)** | 1755 (63.6%) | 701 (61.1%) | 1054 (65.3%) |
| **Age(years), median, (IQR)** | 66 [45 - 79] | 68 [48 - 79] | 65 [44 - 79] |
| **CCI, median, (IQR)** | 0 [0 - 1] | 0 [0 - 1] | 0 [0 - 0] |
| **Antithrombotic medication, n, (%)** | 214 (7.8%) | 97 (8.4%) | 117 (7.3%) |
| **Mechanism of injury, n, (%)** |  |  |  |
| **Pedestrian (traffic accident)** | 498 (18%) | 199 (17.3%) | 299 (18.5%) |
| **Bicycle (traffic accident)** | 214 (7.8%) | 67 (5.8%) | 147 (9.1%) |
| **Motorbike (traffic accident)** | 336 (12.2%) | 145 (12.6%) | 191 (11.8%) |
| **Car (traffic accident)** | 399 (14.5%) | 179 (15.6%) | 220 (13.6%) |
| **Slip** | 187 (6.8%) | 90 (7.8%) | 97 (6%) |
| **Fall** | 917 (33.2%) | 367 (32%) | 550 (34.1%) |
| **Others** | 210 (7.6%) | 101 (8.8%) | 109 (6.8%) |
| **Vital signs, median, (IQR)** |  |  |  |
| **HR (bpm)** | 96 [80 - 116] | 96 [80 - 116] | 96 [80 - 116] |
| **SBP (mmHg)** | 113 [86 - 142] | 110 [84 - 137] | 115 [89 - 144] |
| **RR (/minutes)** | 23 [19 - 29] | 23 [18 - 28] | 23 [19 - 29] |
| **BT (℃)** | 36.2 [35.7 - 36.6] | 36.2 [35.7 - 36.7] | 36.1 [35.7 - 36.6] |
| **GCS, median, (IQR)** | 3 [6 - 14] | 13 [7 - 14] | 12 [6 - 14] |
| **Lactate (mmol/L), median, (IQR)** | 3.33 [2.2 - 5.2] | 3.2 [2.1 - 5.2] | 3.4 [2.3 - 5.2] |
| **AIS, median, (IQR)** |  |  |  |
| Body region 1 (head) | 3 [1 - 5] | 3 [1 - 5] | 4 [1 - 6] |
| Body region 4 (thorax) | 4 [1 - 4] | 4 [1 - 4] | 4 [1 - 4] |
| Body region 5 (abdomen) | 1 [1 - 3] | 1 [1 - 3] | 1 [1 - 3] |
| Body region 8 (lower extremity) | 3 [1 - 5] | 3 [1 - 5] | 3 [1 - 5] |
| **ISS, median, (IQR)** | 26 [22 - 34] | 26 [20 - 34] | 27 [22 - 35] |
| **TRISS, median, (IQR)** | 0.79 [0.45 - 0.92] | 0.82 [0.49 - 0.92] | 0.76 [0.45 - 0.92] |

**IQR: Interquartile range, HR: Heart rate, SBP: Systolic blood pressure, RR: Respiratory rate, BT: Body temperature, GCS: Glasgow Coma Scale, AIS: Abbreviated Injury Scale, ISS: Injury Severity Score, TRISS: Trauma and Injury Severity Score.**

**eTable 5. Treatment details, adverse events, and outcome in validation cohort (2022-2023)**

| **Parameters** | **Total** | **Low FFP group** | **High FFP group** |
| --- | --- | --- | --- |
|  | **N=2761** | **N=1148** | **N=1613** |
| **Transfusion within 24 hours (units), median, (IQR)** |  |  |  |
| RBC | 8 [4 - 12] | 8 [4 - 14] | 6 [4 - 12] |
| FFP | 10 [6 - 18] | 6 [4 - 10] | 14 [8 - 22] |
| PC | 0 [0 - 10] | 0 [0 - 10] | 0 [0 - 10] |
| **FFP-to-RBC ratio, median, (IQR)** | 1.3 [1 - 2] | 1 [0.7 - 1] | 2 [1.4 - 2.5] |
| **Tranexamic acid, n, (%)** | 1552 (56.2%) | 590 (51.4%) | 962 (59.6%) |
| **Surgical intervention, n, (%)** |  |  |  |
| Overall surgical procedure | 2282 (82.7%) | 934 (81.4%) | 1348 (83.6%) |
| Damage control surgery | 452 (16.4%) | 192 (16.7%) | 260 (16.1%) |
| **Adverse event, n, (%)** |  |  |  |
| Overall adverse event | 1101 (39.9%) | 436 (38%) | 665 (41.2%) |
| Pulmonary edema | 21 (0.8%) | 11 (1%) | 10 (0.6%) |
| Pulmonary thromboembolism | 39 (1.4%) | 16 (1.4%) | 23 (1.4%) |
| Acute respiratory distress syndrome | 19 (0.7%) | 9 (0.8%) | 10 (0.6%) |
| Pneumonia | 336 (12.2%) | 137 (11.9%) | 199 (12.3%) |
| Acute Kidney Injury | 75 (2%) | 28 (1.6%) | 47 (2.4%) |
| Sepsis | 75 (2.7%) | 28 (2.4%) | 47 (2.9%) |
| **In-hospital mortality, n, (%)** | 640 (23.2%) | 271 (23.6%) | 369 (22.9%) |

**IQR: Interquartile range, RBC: Red blood cell, FFP: Fresh frozen plasma, PC: Platelet concentrate.**

**eTable 6. Treatment effect in each derived therapeutic target**

|  | **RD (95% CI) of high FFP group** | **NNT (95% CI)** |
| --- | --- | --- |
| **Therapeutic Target 1 (N=722)** |  |  |
| Lactate ≥ 2.66 and BT ≤ 35.9 | -6.7% (-15.1 to 1.7%) | 14.9 (6.6 – 58.5) |
| **Therapeutic Target 2 (N=725)** |  |  |
| SBP ≤ 131 and BT ≤ 35.9 | -6.1% (-13.5 to 1.3%) | 16.4 (7.4-78.5) |
| **Therapeutic Target 3 (N=565)** |  |  |
| GCS ≤ 7 and BT ≤ 35.9 | -5.7% (-14.9 to 3.4%) | 17.5 (6.7 – 29.1) |
| **Therapeutic Target 4 (N=664)** |  |  |
| Lactate ≥ 4.60 and SBP ≤ 131 | -9.6% (-17.8 to -1.5%) | 10.4 (5.6 – 65.3) |
| **Therapeutic Target 5 (N=493)** |  |  |
| GCS ≤ 7 and SBP ≤ 131 mmHg | -9.0% (-19.1 to -1.1% ) | 11.1 (5.2 – 91.5) |

**BT: Body temperature, CI: Confidence interval, GCS: Glasgow Coma Scale, NNT: Number needed to treat, RD: Risk difference, SBP: Systolic blood pressure.**

**eTable 7. Patient characteristics in the target subgroup (GCS ≤12 and lactate ≥4.5 mmol/L) in the validation cohort**

| **Characteristics** | **Target subgroup** |
| --- | --- |
|  | **N=** **571** |
| **Sex (male), n, (%)** | 369 (64.6%) |
| **Age(years), median, (IQR)** | 58 [37 - 75] |
| **CCI, median, (IQR)** | 0 [0 - 0] |
| **Antithrombotic medication, n, (%)** | 35 (6.1%) |
| **Mechanism of injury, n, (%)** |  |
| **Pedestrian (traffic accident)** | 102 (17.9%) |
| **Bicycle (traffic accident)** | 36 (6.3%) |
| **Motorbike (traffic accident)** | 51 (8.9%) |
| **Car (traffic accident)** | 66 (11.6%) |
| **Slip** | 235 (41.2%) |
| **Fall** | 36 (6.3%) |
| **Others** | 45 (7.9%) |
| **Vital signs, median, (IQR)** |  |
| **HR (bpm)** | 112 [91 - 132] |
| **SBP (mmHg)** | 96 [70 - 132] |
| **RR (/minutes)** | 24 [18.5 - 30] |
| **BT (℃)** | 35.9 [35.4 - 36.4] |
| **GCS, median, (IQR)** | 6 [3 - 9] |
| **Lactate (mmol/L), median, (IQR)** | 6.7 [5.5 - 9.4] |
| **AIS, median, (IQR)** |  |
| Body region 1 (head) | 4 [1 - 6] |
| Body region 4 (thorax) | 4 [1 - 5] |
| Body region 5 (abdomen) | 1 [1 - 3] |
| Body region 8 (lower extremity) | 3 [1 - 5] |
| **ISS, median, (IQR)** | 33 [25 - 41] |
| **TRISS, median, (IQR)** | 0.45 [0.15 - 0.69] |

**IQR: Interquartile range, HR: Heart rate, SBP: Systolic blood pressure, RR: Respiratory rate, BT: Body temperature, GCS: Glasgow Coma Scale, AIS: Abbreviated Injury Scale, ISS: Injury Severity Score, TRISS: Trauma and Injury Severity Score.**

**eFigure 1. ﻿** **Group-specific ATE estimates across CATE quintiles using the AIPW estimator.**

This figure shows the estimated group-specific ATE by quintile of CATE ranking obtained from a causal forest model. The horizontal axis indicates the CATE-based quintile ranking (Q1–Q5), where Q1 represents the group with the highest predicted treatment effect and Q5 the lowest. The vertical axis represents the estimated ATE within each group. Red points represent estimates based on the AIPW method. Error bars denote 95% confidence intervals. Model calibration was confirmed by best linear prediction analysis, in which the coefficient of the mean causal forest prediction was 1.00 (p = 0.001), and that of the differential forest prediction (out-of-bag) was 0.84 (p < 0.001), suggesting that the model was well calibrated and effectively captured treatment effect heterogeneity.

**AIPW: augmented inverse probability weighting, ATE: Average treatment effect, CATE: Conditional average treatment effect, OLS: ordinary least squares, Q: quintile.**

**eFigure 2. Variable importance in the causal forest model.**

This figure shows the relative importance of candidate variables in the causal forest model used to estimate the CATE of high FFP transfusion. Variable importance was calculated based on the frequency and quality of splits across trees in the forest.

**Bt: Body temperature, CATE: Conditional average treatment effect, gcs: Glasgow Coma Scale, sbp: Systolic blood pressure, lactate_mmol: Lactate (mmol/L).**

**eFigure 3. Exploratory heatmaps of treatment effect using lactate level, GCS, SBP, and BT**

(A) Heatmaps of mean CATE for in-hospital mortality across subgroups defined by combinations of lactate level, GCS, SBP, and BT, each categorized into tertiles. Darker blue colors indicate greater estimated mortality reduction associated with high FFP-to-RBC ratio transfusion. Values in each cell represent the mean CATE and standard deviation.

(B) Heatmaps of subgroup ATE estimated using IPTW for the same variable combinations. Each cell shows the estimated risk difference and corresponding 95% confidence interval. Darker blue colors represent greater treatment benefit.

**
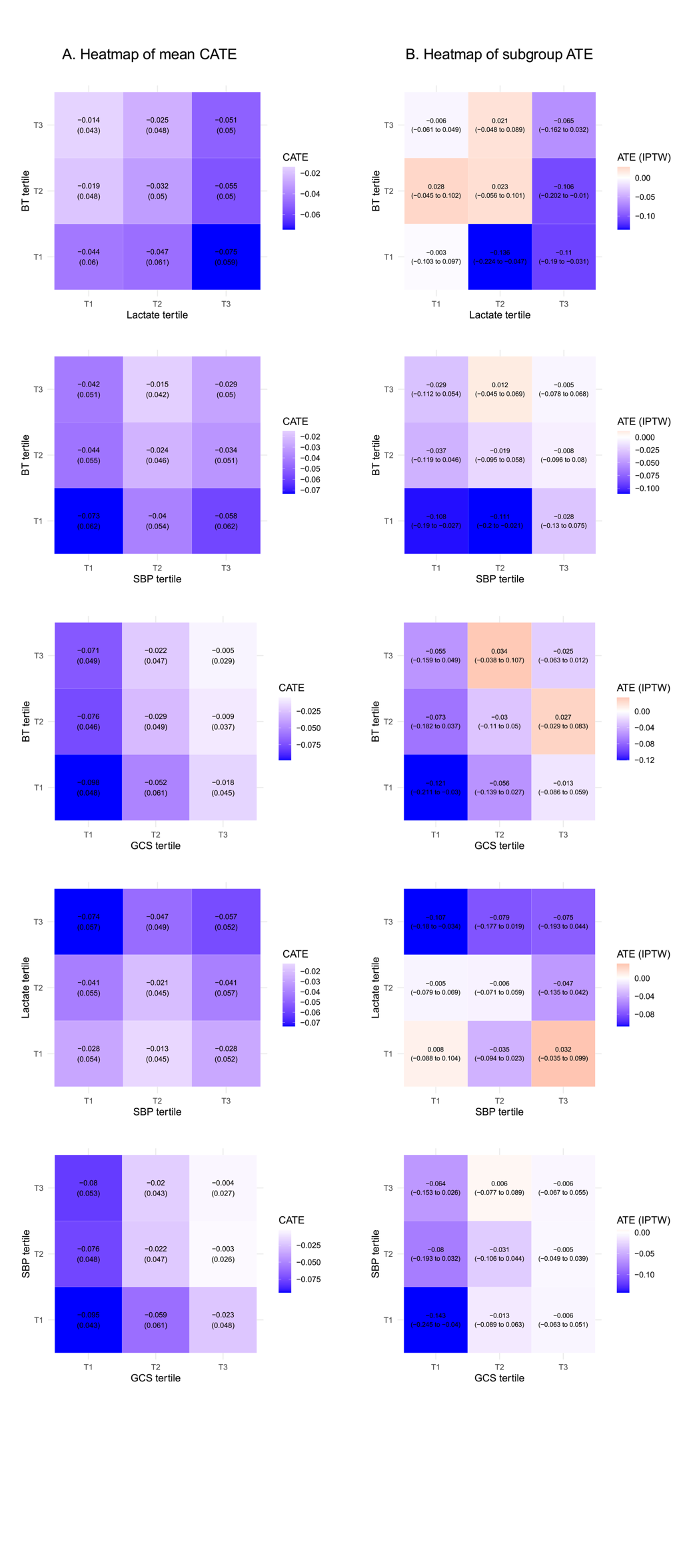
**

**ATE: Average treatment effect, BT: Body temperature, CATE: Conditional average treatment effect, FFP: Fresh frozen plasma, GCS: Glasgow Coma Scale, IPTW: Inverse probability treatment weighting, SBP: Systolic blood pressure.**

**eFigure 4. Subgroup heatmaps of treatment effect using lactate level and GCS.**

Heatmap of mean CATE for in-hospital mortality across subgroups defined by combinations of lactate level, and GCS, each categorized into tertiles. Darker blue colors indicate greater estimated mortality reduction associated with high FFP transfusion. Values in each cell represent the mean CATE and standard deviation.

**CATE: Conditional average treatment effect, FFP: Fresh frozen plasma, GCS: Glasgow Coma Scale.**

**eFigure 5. Flowchart of patient selection for the validation cohort used in sensitivity analyses.**

**
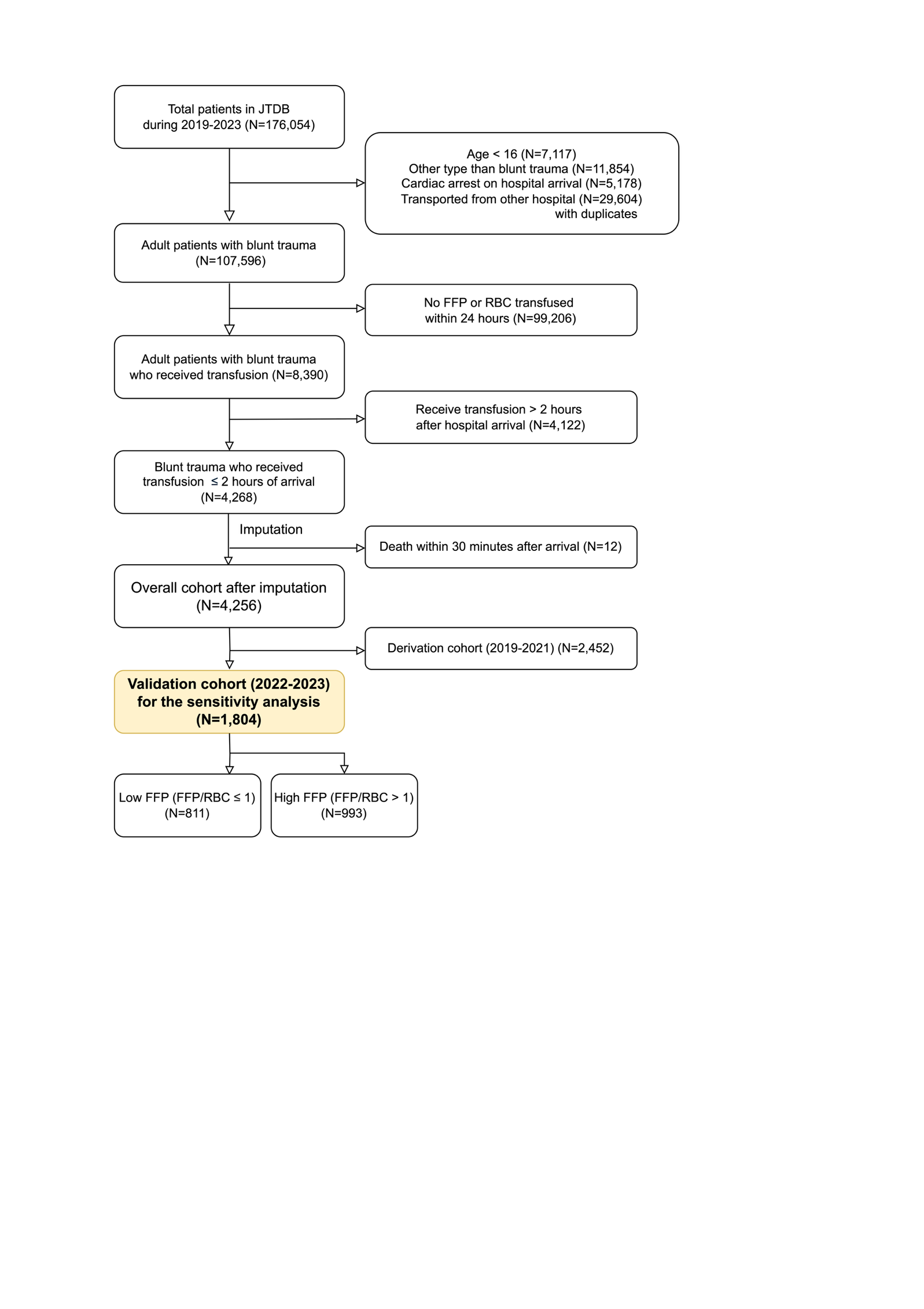
**

**JTDB: Japan Trauma Data Bank, AIS: Abbreviated Injury Scale, ISS: Injury Severity Score, FFP: Fresh frozen plasma, RBC: Red blood cell.**

**eFigure 6. Kaplan–Meier survival curves of patients in the overall and therapeutic target population of the validation cohort.**

Shown are IPTW adjusted Kaplan–Meier survival curves of patients in the overall cohort and the predefined therapeutic target subgroup (lactate ≥ 4.5 mmol/L and GCS ≤ 12) of the validation cohort, after excluding 55 patients with missing survival time information.

**
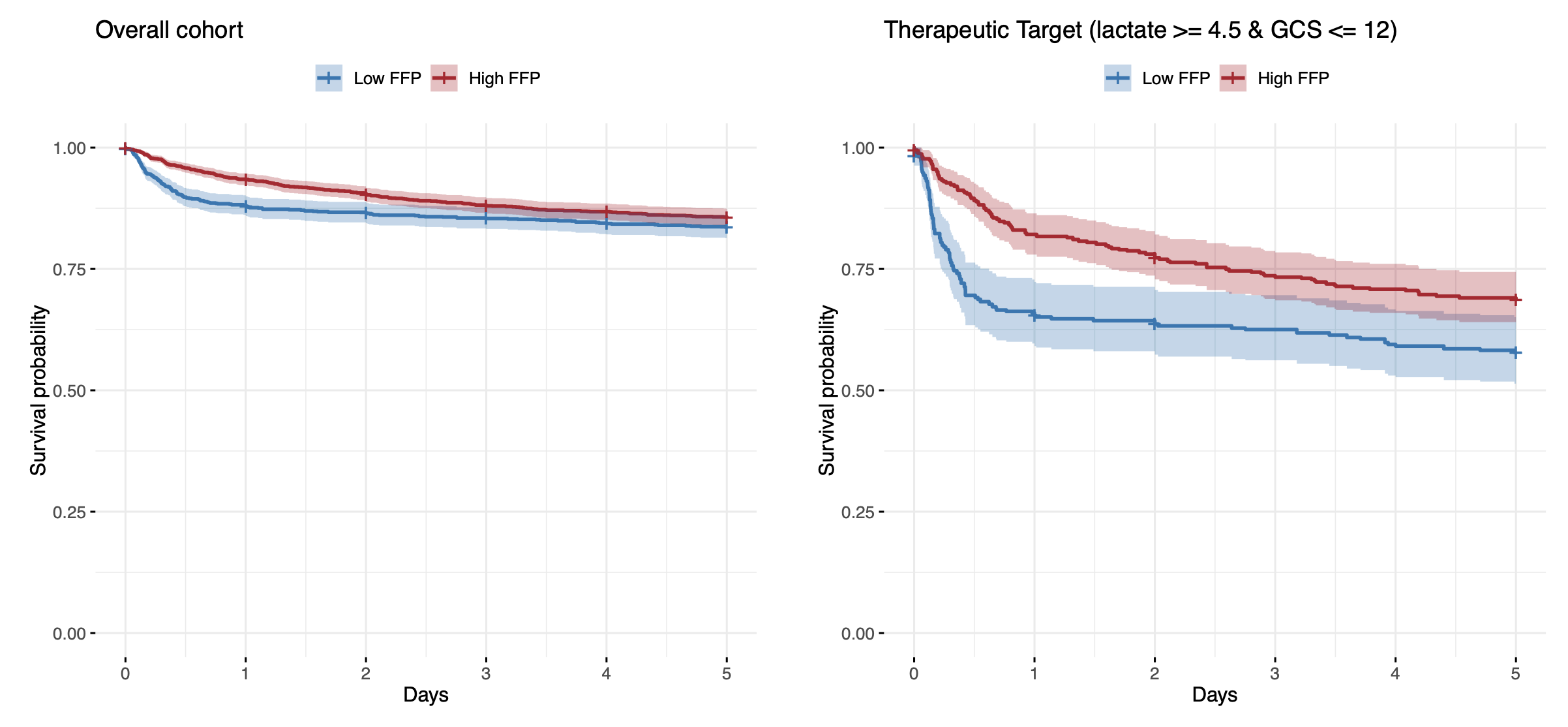
**

**FFP: Fresh frozen plasma, GCS: Glasgow Coma Scale, IPTW: Inverse probability treatment weighting**

**References**

1. Stekhoven DJ, Bühlmann P. MissForest--non-parametric missing value imputation for mixed-type data. Bioinformatics. 2012;28:112–8.

2. Waljee AK, Mukherjee A, Singal AG, Zhang Y, Warren J, Balis U, et al. Comparison of imputation methods for missing laboratory data in medicine. BMJ Open. 2013;3:1–7.

3. Charlson ME, Pompei P, Ales KL, MacKenzie CR. A new method of classifying prognostic comorbidity in longitudinal studies:  development and validation. J Chronic Dis. 1987;40:373–83.
